# Attitudes toward Bisexual Men and Women among a Nationally Representative Probability Sample of Adults in the United States

**DOI:** 10.1371/journal.pone.0164430

**Published:** 2016-10-26

**Authors:** Brian Dodge, Debby Herbenick, M. Reuel Friedman, Vanessa Schick, Tsung-Chieh (Jane) Fu, Wendy Bostwick, Elizabeth Bartelt, Miguel Muñoz-Laboy, David Pletta, Michael Reece, Theo G. M. Sandfort

**Affiliations:** 1 Center for Sexual Health Promotion, School of Public Health, Indiana University, Bloomington, Indiana, United States of America; 2 Graduate School of Public Health, University of Pittsburgh, Pittsburgh, Pennsylvania, United States of America; 3 University of Texas Health Science Center, Houston, Texas, United States of America; 4 Department of Health Systems Science, School of Nursing, University of Illinois, Chicago, Illinois, United States of America; 5 School of Social Work, Temple University, Philadelphia. Pennsylvania, United States of America; 6 Department of Psychiatry, College of Physicians & Surgeons, Columbia University, New York, New York, United States of America; Knox College, UNITED STATES

## Abstract

As bisexual individuals in the United States (U.S.) face significant health disparities, researchers have posited that these differences may be fueled, at least in part, by negative attitudes, prejudice, stigma, and discrimination toward bisexual individuals from heterosexual and gay/lesbian individuals. Previous studies of individual and social attitudes toward bisexual men and women have been conducted almost exclusively with convenience samples, with limited generalizability to the broader U.S. population. Our study provides an assessment of attitudes toward bisexual men and women among a nationally representative probability sample of heterosexual, gay, lesbian, and other-identified adults in the U.S. Data were collected from the 2015 *National Survey of Sexual Health and Behavior* (*NSSHB*), via an online questionnaire with a probability sample of adults (18 years and over) from throughout the U.S. We included two modified 5-item versions of the *Bisexualities*: *Indiana Attitudes Scale (BIAS)*, validated sub-scales that were developed to measure attitudes toward bisexual men and women. Data were analyzed using descriptive statistics, gamma regression, and paired t-tests. Gender, sexual identity, age, race/ethnicity, income, and educational attainment were all significantly associated with participants' attitudes toward bisexual individuals. In terms of responses to individual scale items, participants were most likely to “neither agree nor disagree” with all attitudinal statements. Across sexual identities, self-identified other participants reported the most positive attitudes, while heterosexual male participants reported the least positive attitudes. As in previous research on convenience samples, we found a wide range of demographic characteristics were related with attitudes toward bisexual individuals in our nationally-representative study of heterosexual, gay/lesbian, and other-identified adults in the U.S. In particular, gender emerged as a significant characteristic; female participants’ attitudes were more positive than male participants’ attitudes, and all participants’ attitudes were generally more positive toward bisexual women than bisexual men. While recent population data suggest a marked shift in more positive attitudes toward gay men and lesbian women in the general population of the U.S., the largest proportions of participants in our study reported a relative lack of agreement or disagreement with all affective-evaluative statements in the *BIAS* scales. Findings document the relative lack of positive attitudes toward bisexual individuals among the general population of adults in the U.S. and highlight the need for developing intervention approaches to promote more positive attitudes toward bisexual individuals, targeted toward not only heterosexual but also gay/lesbian individuals and communities.

## Introduction

Bisexual individuals commonly report experiencing stigma, prejudice, and discrimination regarding their sexual identity from both heterosexual and gay/lesbian individuals [1, 2]. This stigmatization of bisexuality among monosexual (i.e., exclusively heterosexual and exclusively homosexual) individuals has been documented in scientific and community literature as “biphobia” (or more recently “binegativity” or “anti-bisexual prejudice”) [3–5]. Biphobia derives in part from heterosexist reaction to sexual expression that is not heteronormative (same-gender sexual expression), as well as monosexist reaction to sexual expression that is not monosexual (e.g., sexual expression with more than one gender). Beyond default assumptions that privilege heterosexuality, negative attitudes toward bisexual groups are also grounded in monosexism, or the belief that people can only be either heterosexual or gay/lesbian, i.e., one or the other. Binary conceptions of sexuality and sexual identity can further fuel dismissive and denigrating attitudes toward bisexual persons, from both heterosexual and gay/lesbian groups [4].

Thus, while sexual minority persons do share some collective experiences of prejudice living in a heteronormative and heterosexist society (e.g., [6, 7]) bisexual individuals are additionally burdened by negative attitudes from gay and lesbian individuals. The deployment of oppressive structures often create multiple levels of oppressive dynamics, from the dominant group to the minority group, and also from minority group to other minority groups [8]. In other words, from a sexual rights perspective, negative attitudes from gay men and lesbian women toward bisexual individuals can be conceptualized as reinforcing of larger oppressive structures against non-heteronormative, non-monosexual forms of expression [9, 10]. Anderson & McCormack note ten forms of “bisexual burden” that may contribute to an increased burden of mental and other health challenges faced by bisexual individuals [11]. Prior research on social attitudes toward sexual minority individuals typically aggregate “lesbian, gay, and bisexual individuals” into a single “LGB” or “sexual minority” category, despite the distinct anti-bisexual prejudices faced by bisexual individuals. Among these, bisexuality is often trivialized as an illegitimate and transitional “phase” in which the person is assumed to be on their way to a “valid” (i.e., monosexual) identity—resulting in colloquial microaggressions such as “bi now, gay later” [5, 12–17]. Bisexual individuals are also commonly characterized as sexually promiscuous, even responsible for serving as a “bridge population” for HIV and other sexually transmitted infections (STI) from partners of one gender to others [3, 18–20]. This is also related to a broader discourse on any form of non-monogamy as “risky” in terms of HIV/STI. As such, another common stereotype involves equating bisexuality with non-monogamy, and bisexual individuals being inherently “incapable” of maintaining monogamous relationships [1, 5].

### Impacts of Negative Attitudes on Health

Negative attitudes derived from such stereotypes may impact the health of bisexual individuals, as they develop into and are enacted in subsequent forms of stigma and prejudice which cumulatively contribute to a state of “minority stress” [21]. The deleterious effects of stigma and prejudice on the health of sexual minority individuals have been well-documented across both physiological and psychological domains. Theoretical models on relations between health disparities and poor health outcomes assume that sexual minority individuals experience distinct elevated levels of stress due to the social stigmatization of their sexual orientation/identity [21, 22]. Broadly, stress can be conceptualized as a biopsychosocial process occurring at three interconnected levels: 1) *biological* (e.g., elevated diastolic blood pressure); 2) *psychological* (e.g., stress affect); and 3) *social* (e.g., experiences of prejudice) [23]. Such a model demonstrates intersections between an individual’s social interactions and their physiological health may be a contributing factor in explaining the highest rates of many adverse health outcomes are found among bisexual individuals, who experience distinct and multiple levels of sexuality-related stigmatization.

Research on the etiologies of health disparities among sexual minority individuals, beneath the surface of “minority stress,” has also highlighted the importance of *social support* and *material resources* as moderators of stigma-induced stress [22]. Bisexual individuals’ experiences of biphobia and subsequent marginalization from gay/lesbian, and heterosexual communities (i.e., sources of social support and resources) place them at elevated risk for physical and mental health disparities [24]. Recent research has documented the existence of a wide range of distinct health disparities among bisexual individuals, relative to their exclusively heterosexual and homosexual counterparts [25, 26]. Compared to their gay and lesbian peers, bisexual individuals report higher rates of mood and anxiety disorders [27, 28], physical and emotional abuse [29], and substance abuse [30], which are strong predictors of early and excess mortality.

### Previous Explorations of Attitudes toward Bisexuality in the United States

Data from large polling agencies reveal that attitudes toward “homosexuality” and “gays and lesbians” have become markedly more positive over the past decade [31], both in the U.S. and throughout much of the world [32]. Previous social and behavioral science researchers have made efforts to assess attitudes toward bisexuality, as distinct from heterosexuality and heterosexuality, in a range of convenience samples in the U.S. and other contexts [33]. Mohr and Rochlen developed the *Attitudes Regarding Bisexuality Scale* (*ARBS*) with convenience samples of lesbian, gay, and heterosexual college students and found two factors associated with attitudes towards bisexuality: tolerance and stability [34]. Tolerance determined whether bisexuality was viewed as morally acceptable, whereas stability determined whether bisexuality was perceived as a legitimate sexual orientation. Attitudes regarding bisexuality varied greatly in respect to the gender of the bisexual individual; specifically, bisexual men were rated less positively than bisexual women in studies with other convenience samples of heterosexual college students [18, 35–37]. Brewster and Moradi developed and psychometrically evaluated another measure, the *Anti-Bisexual Experiences Scale*, using a different scaling approach based on data from convenience samples of bisexual individuals [5]. Their scale development process involved separate measurements of anti-bisexual experiences directed from heterosexual and gay/lesbian individuals, and their findings align with negative attitudinal findings from other samples [1, 34].

Herek [38] conducted the only prior study using probability sampling to examine adults’ attitudes towards bisexual men and women in the U.S., although his sample was limited to heterosexual individuals. Attitudes towards bisexual individuals were less favorable than a wide range of religious, racial, political, and sexuality groups (e.g., Jewish individuals, Haitian individuals, pro-choice individuals, lesbian and gay individuals). Indeed, injection drug users were the only group to be perceived more negatively. Gender differences were observed among heterosexual respondents, with female participants rating bisexual men and women less favorably than gay/lesbian individuals, and male participants reporting more positive views towards lesbian and bisexual women than gay and bisexual men. Although strengthened by probability sampling, the study was limited in that it focused solely on heterosexual individuals’ attitudes (not those of gay/lesbian individuals’). Additionally, the findings are nearly two decades old and predate a number of structural and societal changes that have reflected changes in attitudes among heterosexual individuals toward sexual minority individuals in some ways, including legislation recognizing the legality of same-sex marriage [39].

### Study Aims

Our study aimed to examine attitudes toward bisexual men and women in the general population of the U.S., using a probability sample of heterosexual, gay, lesbian, and other-identified participants from the 2015 *National Survey of Sexual Health and Behavior* (*NSSHB)*. We used abridged versions of the *Bisexualities*: *Indiana Attitudes Scale* (*BIAS*) [40], incorporating two forms measuring distinct attitudes towards bisexual men and bisexual women—i.e., the *BIAS-m a*nd *BIAS-f*, respectively. (While numerous gender identities exist outside a male/female binary, the *BIAS* specifically measures attitudes towards bisexual individuals who identify as men and women without specific mention of whether these men/women are transgender or cisgender, as prior studies have documented differences in attitudes between these two groups.)

The *BIAS* assesses a number of domains that have emerged in previous studies, including bisexual men and women being confused or in transition regarding their sexual orientation, bisexual men and women as hypersexual, and bisexual men and women as vectors of STI. The current study is innovative not only in terms of sampling, by relying on a large probability sample of adults from throughout the U.S., but also because it is among the first to examine attitudes toward bisexual men and women in a nationally-representative sample of not only heterosexual but also gay, lesbian, and other-identified individuals.

## Materials & Methods

The *National Survey of Sexual Health and Behavior* (*NSSHB*) is an ongoing population-based survey of adults and adolescents in the U.S. [41]. The first wave of data was collected in 2009, and subsequently in 2012, 2013, 2014, and 2015. This paper presents the data from the 2015 NSSHB, which was conducted during November and December 2015, via the KnowledgePanel of GfK Research (GfK) (Menlo Park, California). Research panels accessed through GfK are based on a national probability sample established using both random digit dialing (RDD) and an address-based sampling (ABS) frame. ABS involves the probability sampling of a frame of residential addresses derived from the U.S. Postal Service’s Delivery Sequence File, a system that contains detailed information on every mail deliverable address in the U.S. Collectively, the sampling frame from which participants are recruited covers approximately 98% of all U.S. households. Randomly selected addresses are recruited to the research panel through a series of mailings and subsequently by telephone follow-ups to non-responders when possible. To further correct sources of sampling and non-sampling error, study samples are corrected with a post-stratification adjustment using demographic distributions from the March 2015 Current Population Survey (CPS), the monthly population survey conducted by the U.S. Bureau of the Census considered to be the standard for measuring demographic and other trends in the U.S. These adjustments result in a panel base weight that was employed in a probability proportional to size selection method for establishing the samples for this study. Oversampling of specific subgroups are corrected by adjusting the corresponding weights accordingly with the CPS benchmarks serving as reference points.

Once the sample frame was established, all individuals within that frame received a recruitment message from GfK that provided a brief description of the NSSHB and invited them to participate. A total of 2,999 adults (ages 18+) completed the survey, including 1,320 from the general population, 1,230 from an oversample of 18–34 year-olds, and 156 from an oversample of gay men. Given sufficient participation across sexual identity categories, the oversample of gay men was excluded in this analysis, resulting in a total unweighted sample of 2,843 adults.

Post-stratification weights, provided by GfK, were used to maximize generalizations. The post-stratification weights were produced using an iterative proportional fitting procedure that aligned the study sample to all study benchmark distributions from the March 2015 CPS. All results hereafter presents the weighted data. As we focused on the attitudes of non-bisexual individuals about bisexual persons, 33 self-identified bisexual male and 61 bisexual female participants were intentionally excluded from subsequent analyses. Given the very small number of transgender participants (N = 5), these individuals were also removed the analyses. The weighted total sample resulted in 3,221 adults, with 2,434 from the general population and 787 from the 18–34 year-old oversample. A sensitivity analysis excluding the oversample of 18–34 year-old adults was conducted for all analyses. No significant difference were observed, and therefore, the oversample of 18–34 year-old adults was pooled with adults from the general population.

### Measures

In preparation for survey data collection, we engaged a panel of expert reviewers from a range of both scientific and community-based organizations who reviewed the original *BIAS* scales (26-item *BIAS-m* sub-scale for male participants, 27-item *BIAS-f s*ub-scale for female participants) [1, 40]. Previous studies have demonstrated differences in attitudes toward bisexual women in comparison to bisexual men and, as such, we sought to determine whether this was the case in a national probability sample. A panel of six expert reviewers helped the researchers to identify common themes across scale items in order to identify those that tapped into the most important factors that emerged during prior data analysis [1, 12]. While our pilot study, as well as other studies, showed significant differences in attitudes toward bisexual men and bisexual women, the same 5 themes were selected by reviewers as most relevant for both men and women, specifically:

1. Perceptions of confusion
2. Perceptions of HIV/STI “riskiness”
3. Perceptions of non-monogamy
4. Perceptions of promiscuity
5. Perceptions of bisexuality as temporary

After examining feedback from reviewers, the study team identified the highest ranked items and conducted a second level of review with the expert panel. Reviewers agreed, despite similarity of the scale items, that separate items for attitudes toward bisexual men and bisexual women are necessary in order to capture potentially important gender differences in a probability sample.

Table 1 presents the final scale items for the abridged *BIAS-m* and *BIAS-f* measures. These abridged scale items were compiled for the 2015 wave of *NSSHB* in order to collect data from a nationally representative sample of self-identified heterosexual, gay, lesbian, and other participants. The Institutional Review Board of the Human Subjects Office at Indiana University—Bloomington reviewed and approved all study protocols.

**Table 1:** Abridged *Bisexualities*: *Indiana Attitudes Scale* (*BIAS)* items.

**A. Abridged *BIAS-f* [7 point Likert response scale SA-SD]**
*Please respond to the next series of items in terms of the extent that you agree with the following statements about bisexual women (i*.*e*., *those with the capacity for physical*, *romantic*, *and/or sexual attraction to more than one sex or gender)*.
1. I think bisexual women are confused about their sexuality.
2. People should be afraid to have sex with bisexual women because of HIV/STD risks.
3. Bisexual women are incapable of being faithful in a relationship.
4. Bisexual women would have sex with just about anyone.
5. I think bisexuality is just a phase for women.
**B. Abridged *BIAS-m* [7 point Likert response scale SA-SD]**
*Please respond to the next series of items in terms of the extent that you agree with the following statements about bisexual men (i*.*e*., *those with the capacity for physical*, *romantic*, *and/or sexual attraction to more than one sex or gender)*.
1. I think bisexual men are confused about their sexuality.
2. People should be afraid to have sex with bisexual men because of HIV/STD risks.
3. Bisexual men are incapable of being faithful in a relationship.
4. Bisexual men would have sex with just about anyone.
5. I think bisexuality is just a phase for men.

For measures of sexual identity, participants responded to the following item:

Which of the following commonly used terms best describes your sexual orientation?

1. Straight/heterosexual
2. Gay, lesbian, or homosexual
3. Bisexual
4. Asexual (I am not sexually attracted to others)
5. Other, please describe [textbox]

In addition to the widely used identity labels of heterosexual, gay/lesbian, and bisexual, the *NSSHB* offers participants the opportunity to indicate and briefly describe the other identities; in previous probability samples of U.S. adults, the other identity is endorsed by approximately 1% of male and 2% of female respondents [42]. As an increasing number of other identified *NSSHB* participants have described their orientation as asexual, we have offered this term as a separate option in recent waves of data collection. We were particularly interested in examining attitudes among other-identified individuals because, just as diverse individuals are problematically collapsed together under the auspices of “sexual minorities,” a number of non-monosexual identities (e.g., bisexual, pansexual, queer) are sometimes subsumed under the label of “bisexual” [43, 44] and experiences of anti-bisexual prejudice have been found to differ based on the label endorsed by the individual [45].

As anticipated, based on previous waves of *NSSHB* data collection, relatively small numbers of participants self-identified as asexual (N = 21) and other (N = 15). Among the participants who identified as other, participants’ self-reported identities included pansexual, demisexual, gray-asexual, and “I do not identify with any sexual orientation.” A very small number (N = 3) participants did not provide a textbox response for the other item. As the small numbers of asexual and other identified individuals did not provide sufficient statistical power for advanced analyses, we combined these individuals into an aggregate “other” category.

### Statistical Analysis

Differences in sociodemographic characteristics between participants who responded to *BIAS-m* and/or *BIAS-f* were presented descriptively. For an overall measurement of attitudes towards bisexual men and women, we summed the responses for all participants who completed each *BIAS* scale, subtracting by the total number of items answered. *BIAS-m* and *BIAS-f* were also summed to generate a total *BIAS* score among participants who completed both scales. Due to the right-skewness of the scale response distributions, generalized linear modeling with gamma distributions (log link) were utilized to assess the association between sociodemographic characteristics and *BIAS* scores. For each of the three *BIAS* scores, pairwise comparisons by sexual orientation and race/ethnicity, respectively, using t-tests for weighted survey data were conducted. To test for differences between negative attitudes towards bisexual men versus bisexual women, weighted paired t-tests and Wilcoxon signed rank tests were conducted on *BIAS-m* and *BIAS-f* scores. All analyses were performed using Stata version 13 (StataCorp. 2013. Stata Statistical Software: Release 13. College Station, TX: StataCorp LP).

## Results

### Participants

Table 2 provides an overview of the demographic characteristics reported by participants who completed the *BIAS-m* and the *BIAS-f* measurements. Again, self-identified bisexual participants were intentionally excluded from these analyses since the study focused on the attitudes of non-bisexual individuals about bisexual persons. For the male version, most participants in our sample identified as heterosexual or straight (95%, N = 2,885), with a further 3.7% identifying as gay (N = 112) and a remaining 1.2% (N = 36) as other. Proportions were identical for the female version, with slightly more heterosexual participants completing the sub-scale. For the *BIAS-m* scale as a whole, Cronbach’s alpha was 0.911; for the *BIAS-f* scale as a whole, Cronbach’s alpha was 0.909.

**Table 2:** Sociodemographics of participants completing the *Bisexualities*: *Indiana Attitude Scale* (*BIAS)* targeting bisexual males (*BIAS-m*) and bisexual females (*BIAS-f*).

		*BIAS-m* (N = 3,033)	*BIAS-f* (N = 3,046)
*Sociodemographics*		*n (%)*	*n (%)*
Age			
	18–24	348 (11.5)	348 (11.4)
	25–34	589 (19.4)	590 (19.4)
	35–44	443 (14.6)	443 (14.5)
	45–54	507 (16.7)	507 (16.6)
	55+	1,147 (37.8)	1,160 (38.1)
Gender			
	Male	1,437 (47.5)	1,438 (47.3)
	Female	1,591 (52.5)	1,604 (52.7)
Race/Ethnicity			
	White, non-Hispanic	1,983 (65.4)	1,985 (65.2)
	Black, non-Hispanic	358 (11.8)	363 (11.9)
	Other, non-Hispanic	199 (6.6)	199 (6.5)
	Hispanic	446 (14.7)	453 (14.9)
	Multiple races/ethnicities	47 (1.5)	47 (1.5)
Education			
	Less than high school	342 (11.3)	349 (11.4)
	High school	917 (30.3)	917 (30.1)
	Some college	860 (28.4)	860 (28.2)
	Bachelor’s degree or higher	914 (30.1)	920 (30.2)
Household income			
	<$25,000	522 (17.2)	522 (17.2)
	$25,000-$49,999	643 (21.2)	650 (21.3)
	$50,000-$74,999	529 (17.5)	529 (17.4)
	> = $75,000	1,339 (44.1)	1,346 (44.2)
Geographic region			
	Northeast	558 (18.4)	560 (18.4)
	Midwest	649 (21.4)	649 (21.3)
	South	1,128 (37.2)	1,139 (37.4)
	West	698 (23.0)	698 (22.9)
Sexual orientation			
	Heterosexual	2,885 (95.1)	2,898 (95.2)
	Gay/Lesbian	112 (3.7)	112 (3.7)
	Bisexual	-	-
	Other	36 (1.2)	36 (1.2)

### Descriptive Findings

Table 3 provides an overview of the descriptive responses from all participants for each individual scale item on the *BIAS-m* and *BIAS-f* sub-scales. Regarding the response rate for the *BIAS* sub-scales, 97.0% responded to both scales, 2.5% did not respond to either scale, 0.02% responded to *BIAS-m* but not *BIAS-f*, and 0.5% responded to *BIAS-f* but not *BIAS-m*. In comparison to participants who responded consistently to both scales (either both responded or both non-response), the people who responded to one scale and not the other is extremely small (less than 1%). The location of the *BIAS-f* items before the *BIAS-m* items may account for the very slightly higher non-response rate of the *BIAS-m* items.

**Table 3:** *Bisexualities*: *Indiana Attitudes Scale* response distributions for *BIAS-m* and *BIAS f* sub-scales.

***BIAS-m***	Strongly disagree	Disagree	Somewhat disagree	Neither agree nor disagree	Somewhat agree	Agree	Strongly Agree
	*%*	*%*	*%*	*%*	*%*	*%*	*%*
I think bisexual men are confused about their sexuality	12.4	13.5	6.0	34.0	10.2	11.2	12.6
People should be afraid to have sex with bisexual men because of HIV/STD risks	11.5	9.2	6.1	29.4	15.3	13.2	15.0
Bisexual men are incapable of being faithful in a relationship	17.8	15.0	8.5	37.1	7.0	7.2	7.3
Bisexual men would have sex with just about anyone	17.5	14.9	8.1	34.5	8.5	7.2	9.2
I think bisexuality is just a phase for men	18.7	17.1	9.9	37.7	7.4	3.6	5.0
***Bisexualities*: *Indiana Attitudes Scale-Female* (5 Items) response distributions (N = 3,046)**							
***BIAS-f***	Strongly disagree	Disagree	Somewhat disagree	Neither agree nor disagree	Somewhat agree	Agree	Strongly Agree
	*%*	*%*	*%*	*%*	*%*	*%*	*%*
I think bisexual women are confused about their sexuality	12.5	14.4	5.4	35.7	10.4	11.0	10.6
People should be afraid to have sex with bisexual women because of HIV/STD risks	13.8	12.8	7.1	35.0	11.3	9.5	10.1
Bisexual women are incapable of being faithful in a relationship	18.5	17.3	7.5	38.0	7.2	5.1	6.0
Bisexual women would have sex with just about anyone	19.3	17.3	8.1	36.0	7.9	4.3	6.3
I think bisexuality is just a phase for women	17.3	17.2	10.0	38.1	8.7	3.7	4.4

*Bisexualities*: *Indiana Attitudes Scale-Male* (5 Items) response distributions (N = 3,033)

In terms of responses, for both male and female sub-scale items, participants were most likely to “neither agree nor disagree” with attitudinal statements (Table 3). For all items, the proportions of “neither agree nor disagree” responses were over one-third; indeed, nearly 40% neither agreed nor disagreed regarding the capability of bisexual men and/or bisexual women to be “faithful” in a relationship.

### Difference between Overall Attitudes towards Bisexual Men versus Bisexual Women

Average *BIAS* scores for participants completing both scales were 13.64 (95% CI: 13.08–14.20) towards bisexual men and 12.80 (95% CI: 12.25–13.35) towards bisexual women. Negative attitudes towards bisexual men was significantly greater than negative attitudes towards bisexual women (p<0.001 by both weighted paired t-test and Wilcoxon signed rank test).

### Factors Influencing Attitudes toward Bisexual Men and Women

Table 4 presents the relationships between specific demographic characteristics and mean *BIAS* scores. Age emerged as a related factor with attitudes toward bisexual men and women. Participants under the age of 25 years old reported significantly lower *BIAS* scores; thus, younger participants reported more positive attitudes (p<0.001). Gender was also a significant factor, such that, relative to men in the sample, women reported significantly lower scores on the *BIAS-f*. There were not; however, any significant gender differences between participants on the *BIAS-m*.

**Table 4:** The relationship between sociodemographic characteristics and *BIAS* scores towards bisexual males (BIAS-m) and females (BIAS-f).

		BIAS-m			BIAS-f			Total BIAS		
*Variables*		*β*	*SE*	*p*	*β*	*SE*	*p*	*β*	*SE*	*p*
Intercept		2.61	0.03	<0.001	2.47	0.03	<0.001	3.23	0.03	<0.001
Age < 25 years		-0.22	0.04	<0.001	-0.16	0.04	<0.001	-0.19	0.04	<0.001
Gender										
	Male (ref)	0.00	--	--	0.00	--	--	0.00	--	--
	Female	-0.11	0.03	0.101	-0.05	0.03	0.100	-0.08	0.03	0.005
Race/Ethnicity										
	White	0.00	--	--	0.00	--	--	0.00	--	--
	Black	0.15	0.05	0.002	0.13	0.05	0.006	0.14	0.05	0.004
	Other	0.02	0.06	0.700	0.09	0.05	0.079	0.05	0.05	0.314
	Hispanic	0.07	0.04	0.089	0.11	0.04	0.011	0.08	0.04	0.049
	2+ races	0.09	0.08	0.240	0.11	0.07	0.102	0.10	0.07	0.162
High school or less		0.10	0.02	<0.001	0.15	0.02	<0.001	0.12	0.02	<0.001
Income < $25,000		0.18	0.04	<0.001	0.20	0.04	<0.001	0.19	0.04	<0.001
Sexual orientation										
	Heterosexual (ref)	0.00	--	--	0.00	--	--	0.00	--	--
	Gay/Lesbian	-0.59	0.13	<0.001	-0.57	0.18	0.002	-0.57	0.15	<0.001
	Other	-0.66	0.13	<0.001	-0.62	0.12	<0.001	-0.65	0.12	<0.001

Race/ethnicity was significantly related to the participants’ attitudes toward bisexual individuals. In terms of the total *BIAS* measure, White/non-Hispanic participants reported the lowest mean scores (i.e., most positive scores), while Black/non-Hispanic participants reported the highest score (i.e., most negative scores). Similar patterns were found with the male and female sub-scales. As seen in Table 5, significant differences were found between White/non-Hispanic and Black/non-Hispanic participants (p = 0.003), as well as Other/non-Hispanic and Black/non-Hispanic participants. (p = 0.006).

**Table 5:** Estimated mean differences in *BIAS* scores by race/ethnicity.

	White, non-Hispanic	Black, non-Hispanic	Other, non-Hispanic	Hispanic	2+ Races, non-Hispanic	Mean difference	SE	p
BIAS-m score	13.28	15.56	--	--	--	-2.28	0.74	0.003*
	13.28	--	13.03	--	--	0.25	0.73	0.738
	13.28	--	--	13.94	--	-0.67	0.49	0.175
	13.28	--	--	--	14.45	-1.18	1.06	0.272
	--	15.56	13.03	--	--	2.53	0.88	0.006*
	--	15.56	--	13.94	--	1.62	0.71	0.028*
	--	15.56	--	--	14.45	1.11	1.23	0.371
	--	--	13.03	13.94	--	-0.91	0.62	0.145
	--	--	13.03	--	14.45	-1.42	1.34	0.295
	--	--	--	13.94	14.45	-0.51	1.19	0.671
BIAS-f score	12.28	14.45	--	--	--	-2.18	0.74	0.005*
	12.28	--	12.62	--	--	-0.35	0.56	0.535
	12.28	--	--	13.50	--	-1.22	0.50	0.019*
	12.28	--	--	--	14.04	-1.77	0.94	0.065
	--	14.45	12.62	--	--	1.83	0.83	0.032*
	--	14.45	--	13.50	--	0.95	0.72	0.193
	--	14.45	--	--	14.04	0.41	1.21	0.736
	--	--	12.62	13.50	--	-0.88	0.59	0.141
	--	--	12.62	--	14.04	-1.42	1.11	0.208
	--	--	--	13.50	14.04	-0.54	1.10	0.623
Total BIAS score	25.53	29.82	--	--	--	-4.30	1.46	0.005*
	25.53	--	25.65	--	--	-0.12	1.26	0.922
	25.53	--	--	27.23	--	-1.70	0.99	0.094
	25.53	--	--	--	28.50	-2.97	1.97	0.138
	--	29.82	25.65	--	--	4.17	1.67	0.016*
	--	29.82	--	27.23	--	2.60	1.46	0.081
	--	29.82	--	--	28.50	1.33	2.42	0.586
	--	--	25.65	27.23	--	-1.57	1.18	0.189
	--	--	25.65	--	28.50	-2.84	2.42	0.246
	--	--	--	27.23	28.50	-1.27	2.26	0.577

* p<0.05

Income was significantly related to participants’ attitudes. In particular, participants who earned less than $25,000 annually reported significantly higher *BIAS* mean scores. Thus, higher income participants were more likely to report more positive attitudes toward bisexual men and women. Similarly, educational attainment was significantly associated with participants' attitudes. Participants whose educational attainment was a high school diploma or less reported significantly higher *BIAS* scores. Thus, participants with higher levels of educational attainment were more likely to report more positive attitudes toward bisexual men and women.

Last, sexual identity was also significantly related to participants’ attitudes. Specifically, other-identified participants reported the lowest *BIAS* scores, followed by gay/lesbian participants and lastly heterosexual participants. Table 6 demonstrates relationships among various sexual identity categories for both *BIAS-m* and *BIAS-f* measures. Overall, sexual minority individuals (other and gay/lesbian) reported significantly more positive attitudes toward bisexual men and women than heterosexual participants. This was finding was consistent for both subscales on attitudes toward bisexual men and women.

**Table 6:** Estimated mean differences in *BIAS* scores by sexual orientation.

	Heterosexual	Gay/Lesbian	Other	Mean difference	SE	P
*BIAS-m* score	13.94	8.04	--	5.91	0.96	<0.001*
	13.94	--	7.05	6.90	0.95	<0.001*
	--	8.04	7.05	0.99	1.48	0.507
*BIAS-f* score	13.05	7.40	--	5.65	1.18	<0.001*
	13.05	--	6.93	6.11	0.93	<0.001*
	--	7.40	6.93	0.47	1.63	0.775
Total *BIAS* score	26.92	15.43	--	11.49	2.13	<0.001*
	26.92	--	13.90	13.03	1.84	<0.001*
	--	15.43	13.90	1.54	3.08	0.619

* p<0.05

## Discussion

Our findings are among the first to document attitudes toward bisexual men and women among a probability sample of adults in the general population of the U.S. Our data from the 2015 *NSSHB* provide a snapshot of attitudes toward bisexual men and women in the U.S. that is both nationally representative and inclusive not only of self-identified heterosexual individuals but also, importantly, among individuals of diverse sexual identities. While a small number of studies have examined attitudes toward bisexual individuals (or bisexuality) in a range of convenience samples, our paper is only the second that explores such attitudes in a nationally representative sample, and is the first to do so in a sample of gay, lesbian, and other-identified individuals. In the sole prior probability study of heterosexual individuals’ attitudes toward bisexual men and women, self-reported attitudes (toward bisexual men, in particular) were extremely negative [38]. As such, our findings represent a “shift” in attitudes toward bisexual men and women from negative to more neutral in the general population. Further nationally representative data on attitudes toward bisexual men and women are needed in order to provide a clearer sense of change over time in such attitudes.

Positive attitudes toward gay and lesbian individuals among heterosexual individuals have dramatically increased over the past decade [31], both in the U.S. and around the world [32]. In the U.S., every demographic group has seen an increase in acceptance of gay and lesbian individuals including religious groups, all political parties, and young people [46]. These rates have remained constant almost a year after the Supreme Court ruling Obergefell v. Hodges [46]. However, in terms of attitudes toward bisexual men and women, the majority of participants in our study remained “middle of the road” and were most likely to report neither agreeing nor disagreeing with the range of sentiments and stereotypes embedded within the *BIAS* items. These responses can be interpreted in a variety of ways, including the participant not knowing, the participant being unsure, or the participant not caring [47]. Additionally, mid-point responses may also reflect social desirability bias [48]. While it is encouraging that most participants did not report more explicitly negative attitudes, that over one-third of participants neither agreed nor disagreed with every scale item may also be indicative of a general ambivalence toward bisexual men and women within the general population [49]. This may be reflective of larger cultural shifts away from expressing explicitly negative or discriminatory attitudes toward minority groups, while implicit or unconscious biases may still operate [50]. This may also be partially due to the relative invisibility of bisexual individuals and positive bisexual role models, relative even to increasing portrayals of gay men and lesbians in media and social consciousness [3, 28]. It may also reflect respondents’ unfamiliarity with bisexual people as bisexual men and women are significantly less likely than gay and lesbian individuals to disclose their identities and behaviors to friends and family members [51, 52].

In terms of specific scale items, the most commonly endorsed belief was that bisexual men and women are at a heightened risk for HIV/STI. This finding is in line with prior assessments of attitudes toward bisexual men and women in convenience samples in terms of “sexual riskiness” [1], as well as descriptions of “sexual irresponsibility” as a commonly reported anti-bisexual experience among bisexual individuals [5]. This sentiment is likely an artifact from the early days of the HIV epidemic, in which bisexual men, in particular, were stereotyped as a “bridge population” between their male partners and their presumably unaware female partners [53, 54]. [1, 5] However, recent research has demonstrated that, in the U.S., the number of bisexual men who are HIV-positive is likely no greater than the number of heterosexual men who are HIV-positive [55]; that bisexual men who are HIV-positive report lower proportions of female sexual partners, and lower rates of sexual intercourse with women, than bisexual men who are HIV-negative [56]; and that Black men who are gay-identified but behave bisexually are more likely to use HIV pre-exposure prophylaxis (PrEP) than Black gay men who do not report sex with women [52]. Taken together, these findings suggest that social discourse about bisexual men’s viral bridging behavior does not take into account current strategies that bisexual men have developed to minimize risk to themselves and their partners, whether male, female, or other. Additionally, while it is important to document the existence of health disparities, researchers and public health professionals are encouraged to be mindful of their role in perpetuating these stereotypes. Researchers are encouraged to engage with bisexual communities in both research design and dissemination in order to ensure that their research does not cause further unintentional damage to these communities [24].

Several findings on demographic factors associated with self-reported attitudes are parallel to results from prior studies focused on convenience samples and warrant further discussion [1, 33]. Gender was significantly associated with self-reported attitudes. Specifically, women were more likely to report more positive attitudes for bisexual men and women. These findings are consistent with the larger body of research on attitudes toward “LGB” populations, wherein women consistently demonstrate more positive attitudes toward sexual and gender minority groups than men [38, 57]. This may be because, overall, women tend to be less conservative and look more favorably upon social issues related to equality [58]. Similarly, attitudes toward sexual minority women are less negative than those towards sexual minority men. Worthen offers a number of explanations for this “gender gap”, including the sexualization of bisexual and lesbian women [59]. As it pertains to our findings, we would also posit that society constantly monitors women’s sexual behavior and uses that behavior to define and control women (e.g., being labelled as a “slut” versus a “prude”) [60]. Being made aware of the damage that this constant regulation may have on the way that they are able to experience their life may make women less prone to posing similar regulations on the lives of others. Additionally, more women identify as bisexual than men and women have also been thought to be more sexually fluid than men [14]. It is possible that women are less judgmental about bisexuality because many have considered their own bisexuality and/or sexual fluidity. Additionally, as men are traditionally expected to more rigidly conform to gender explicitly heteronormative norms and stereotypes, their attitudes may be markedly less “fluid” than women’s [61], though attitudes about stringent masculinity expectations are changing rapidly in youth populations in the U.S. and elsewhere [62].

Age also emerged as significant, specifically that lower age (under 25 years) was associated with more positive attitudes. These findings are consistent with recent qualitative cohort research on bisexual men, in which those in the 18–24 year old age cohort reported more acceptance from their heterosexual and gay and lesbian peers, than those in the older cohorts [63]. Contemporary youth are more likely to consider and define their sexuality and gender in ways that are less rigid than older generations. This is reflected both in national surveys that have found an increasing number of youth adopting non-monosexual identity labels (e.g., bisexual, pansexual, queer) and further highlighted by the number of young celebrities who have publicly claimed similar labels [64]. Youth may be more sensitive and resistant to stereotypes about those who adopt a bisexual label based on reflections of their own sexualities.

In relation to sexual identity, this may also explain why other-identified participants were more likely than others to report more positive attitudes toward bisexual men and women. Along the same lines as above, those who have questioned their identity and do not define within ‘traditional’ categories may relate to and be more sensitive about similar stereotypes. It is positive that gay/lesbian-identified individuals responded with less negative attitudes toward bisexual men and women than heterosexual participants. However, their attitudes were also less positive than other-identified individuals. This may be further evidence that nominally inclusive “LGB” support mechanisms may not be as relevant to bisexual individuals as they are to gay men and lesbian women [1]. Additionally, regarding sexual prejudice more broadly, oppressive structures operate through multiple levels, and to a large extent monosexism might be a fundamental source of oppressive attitudes faced by bisexual individuals. It is important to also consider that monosexism is dominated by heteronormative discourse thus, placing non-heterosexual forms of expression in antithesis to heteronormativity but also in conflict with each other.

In relation to race/ethnicity, Black/non-Hispanic participants were most likely to report more negative attitudes toward bisexual men and women. This is interesting given that, in many convenience samples of gay and bisexual men, Black men are significantly more likely to identify as bisexual than as gay [53, 65]. More negative attitudes among Black participants may also be the result of recent sensationalized negative media portrayals of Black behaviorally bisexual men “on the down low,” which has been alluded to in prior qualitative research [53]. In our pilot research, biracial/multiracial participants reported more positive attitudes toward bisexual men and women than their counterparts [1]. Null significance may be due to statistical power, due to the relatively smaller number of participants who report 2+ races/ethnicities in our nationally representative sample, and this issue may be explored in future research.

Both educational attainment and income level were associated with participants’ attitudes. Specifically, lower levels of income were reported with less positive attitudes toward bisexual men and women. This was also true of education, in that lower educational attainment was associated with less positive attitudes toward bisexual men and women. When away from home in an academic setting, students are often encouraged to question their beliefs, tolerate and accept diversity, and sometimes explore their sexuality. They may also be exposed to others whose sexual lives differ from their own. This exposure (whether in their lives or to the lives of others) may reduce judgement that they may have had about an otherwise unknown group [2].

### Limitations

As with all social science research, this study is not without limitations. Future research would benefit from exploring attitudes toward bisexual transgender, non-binary, gender-queer, and other groups of individuals who may not identify as male or female. While some perhaps have not included these non-binary identities in previous studies on bisexuality, due to the assumption that the numbers of people who would report these identities is too low, it may be the case that we simply have not afforded individuals the option to report such identities consistently in research.

Additionally, as in prior waves of *NSSHB*, small numbers of individuals self-identified their sexual orientation as other. Unfortunately, we were not able to capture a large enough sub-sample size to explore the diversity among individuals who are captured under the other umbrella (e.g., similarities or differences among asexual, pansexual, and others who do not self-identify their sexual orientation with “traditional” labels, or who do not label themselves at all). An interesting finding that emerged, however, is that attitudes toward bisexual men and women among the aggregated other individuals were the most positive of all sexual identity sub-groups. Future research on sexuality-related attitudes and concerns among other-identified individuals is warranted.

## Conclusions

Although it was not the aim of our study to directly examine previously established health disparities, the pervasive absence of positive attitudes toward bisexual men and women may be one of a number of other complex factors that continue to drive higher rates of adverse health outcomes among bisexual men and women. Researchers should continue to explore these unique health disparities in the context of social attitudes toward bisexual men and women in the U.S. After documenting the existence of an absence of positive attitudes toward bisexual men and women in the general U.S. population, we encourage future research to explore intervention opportunities focused on assessing, understanding, and eliminating biphobia—for example, among clinicians and other service providers—and determining how health disparities among bisexual men and women can be alleviated.

## References

[1] Friedman MR, Dodge B, Schick V, Herbenick D, Hubach R, Bowling J, et al From bias to bisexual health disparities: Attitudes toward bisexual men and women in the United States. LGBT Health. 2014;1:309–18. 10.1089/lgbt.2014.0005 25568885 PMC4283842

[2] Herek GM, McLemore KA. Sexual prejudice. In: Fiske ST, editor. Annual Review of Psychology, Vol 64. Annual Review of Psychology. 642013. p. 309–33. 10.1146/annurev-psych-113011-143826 22994920

[3] Ochs R. Biphobia: It goes more than two ways In: Firestein BA, editor. Bisexuality: The Psychology and Politics of an Invisible Minority Thousand Oaks, CA: Sage Publicaitons; 1996 p. 217–39.

[4] Roberts TS, Horne SG, Hoyt WT. Between a Gay and a Straight Place: Bisexual Individuals’ Experiences with Monosexism. Journal of Bisexuality. 2015;15(4):554–69. 10.1080/15299716.2015.1111183

[5] Brewster ME, Moradi B. Perceived Experiences of Anti-Bisexual Prejudice: Instrument Development and Evaluation. Journal of Counseling Psychology. 2010;57(4):451–68. 10.1037/a0021116

[6] Herek GM. Confronting sexual stigma and prejudice: Theory and practice. Journal of Social Issues. 2007;63(4):905–25. 10.1111/j.1540-4560.2007.00544.x

[7] Herek GM. The psychology of sexual prejudice. Current Directions in Psychological Science. 2000;9(1):19–22.

[8] Freire P. Pedagogy of the Oppressed. London: Bloomsbury Publishing; 2000.

[9] Sidanius J, Pratto F. Social Dominance: An Intergroup Theory of Social Hierarchy and Oppression. Cambridge, UK: Cambridge University Press; 2001.

[10] Bartky SL. Femininity and Domination: Studies in the Phenomenology of Oppression. New York, NY: Routledge; 1990.

[11] Anderson E, McCormack M. The changing dynamics of bisexual men's lives: Social research perspectives. Switzerland: Springer International Publishing; 2016.

[12] Friedman M, Dodge B, Schick V, Goncalves G, Herbenick D, Hubach R, et al., editors. Not a legitimate sexual orientation: Attitudes towards bisexual men and women as risk factor for HIV/AIDS. 141st APHA Annual Meeting and Exposition (November 2-November 6, 2013), APHA Retrieved from https://aphaconfexcom/apha/141am/webprogram/Paper293050html; 2013.

[13] Alarie M, Gaudet S. “I don't know if she is bisexual or if she just wants to get attention”: Analyzing the various mechanisms through which emerging adults invisibilize bisexuality. Journal of Bisexuality. 2013;13(2):191–214.

[14] Diamond LM. Sexual fluidity: Understanding women's love and desire. Cambridge, MA: Harvard University Press; 2008.

[15] Rust PCR. Bisexuality: The state of the union. Annual Review of Sex Research, Vol 13, 2002. 2002;13:180–240. CCC:000184025400005. 12836732

[16] Zivony A, Lobel T. The Invisible Stereotypes of Bisexual Men. Archives of Sexual Behavior. 2014;43(6):1165–76. 10.1007/s10508-014-0263-9. WOS:000340608300017. 24558124

[17] Bostwick W, Hequembourg A. 'Just a little hint': bisexual-specific microaggressions and their connection to epistemic injustices. Culture, health & sexuality. 2014;16(5):488–503. Epub 2014/03/29. 10.1080/13691058.2014.889754 . 24666221

[18] Eliason M. Bi-negativity: The stigma facing bisexual men. Journal of Bisexuality. 2001;1(2–3):137–54.

[19] Rust PCR. Bisexuality: A contemporary paradox for women. Journal of Social Issues. 2000;56(2):205–21.

[20] Spalding LR, Peplau LA. The unfaithful lover: Heterosexuals' perceptions of bisexuals and their relationships. Psychology of Women Quarterly. 1997;21(4):611–25.

[21] Meyer IH. Prejudice, social stress, and mental health in lesbian, gay, and bisexual populations: Conceptual issues and research evidence. Psychological Bulletin. 2003;129:674–97. 10.1037/0033-2909.129.5.674 12956539 PMC2072932

[22] Hatzenbuehler ML, Phelan JC, Link BG. Stigma as a fundamental cause of population health inequalities. American journal of public health. 2013;103(5):813–21. 10.2105/AJPH.2012.301069 23488505 PMC3682466

[23] Hatzenbuehler ML. How does sexual minority stigma “get under the skin”? A psychological mediation framework. Psychological bulletin. 2009;135(5):707 10.1037/a0016441 19702379 PMC2789474

[24] Dodge B, Schnarrs PW, Reece M, Goncalves G, Martinez O, Nix R, et al Community involvement among behaviourally bisexual men in the Midwestern USA: experiences and perceptions across communities. Culture Health & Sexuality. 2012;14(9):1095–110. 10.1080/13691058.2012.721136. WOS:000309703200009. 22978551 PMC3463651

[25] Dodge B, Sandfort TGM. A review of mental health research on bisexual individuals when compared to homosexual and heterosexual individuals In: Firestein BA, editor. Becoming visible: Counseling bisexuals across the lifespan. New York: Columbia University Press; 2007 p. 28–51.

[26] Miller M, André A, Ebin J, Bessonova L. Bisexual Health: An Introduction and Model Practices for HIV/STI Prevention Programming. Washington, D.C: The National Gay and Lesbian Task Force Policy Institute; 2007.

[27] Bostwick WB, Boyd CJ, Hughes TL, McCabe SE. Dimensions of sexual orientation and the prevalence of mood and anxiety disorders in the United States. American Journal of Public Health. 2010;100(3):468–75. 10.2105/AJPH.2008.152942 19696380 PMC2820045

[28] Dodge B, Schnarrs PW, Reece M, Martinez O, Goncalves G, Malebranche D, et al Individual and Social Factors Related to Mental Health Concerns Among Bisexual Men in the Midwestern United States. Journal of Bisexuality. 2012;12(2):223–45. 10.1080/15299716.2012.674862 22745591 PMC3383005

[29] Friedman MS, Marshal MP, Guadamuz TE, Wei C, Wong CF, Saewyc EM, et al A meta-analysis of disparities in childhood sexual abuse, parental physical abuse, and peer victimization among sexual minority and sexual nonminority individuals. American Journal of Public Health. 2011;101(8):1481–94. 10.2105/ajph.2009.190009. WOS:000293149500028. 21680921 PMC3134495

[30] Meyer IH, Dietrich J, Schwartz S. Lifetime prevalence of mental disorders and suicide attempts in diverse lesbian, gay, and bisexual populations. American Journal of Public Health. 2008;98(6):1004–6. 10.2105/AJPH.2006.096826 17901444 PMC2377299

[31] Gallup. Gay and Lesbian Rights 2015 [cited 2016 June 13]. Available: http://www.gallup.com/poll/1651/gay-lesbian-rights.aspx.

[32] Pew Research Center. The Global Divide on Homosexuality 2013 [cited 2016 June 13]. Available: http://www.pewglobal.org/2013/06/04/the-global-divide-on-homosexuality/.

[33] Israel T, Mohr JJ. Attitudes Toward Bisexual Women and Men. Journal of Bisexuality. 2004;4(1–2):117–34. 10.1300/J159v04n01_09

[34] Mohr JJ, Rochlen AB. Measuring attitudes regarding bisexuality in lesbian, gay male, and heterosexual populations. Journal of Counseling Psychology. 1999;46:353–69. 10.1037/0022-0167.46.3.353

[35] Eliason MJ. The prevalence and nature of biphobia in heterosexual undergraduate students. Archives of Sexual Behavior. 1997;26(3):317–26. 10.1023/a:1024527032040. WOS:A1997WX11800005. 9146816

[36] Worthen MGF. An Argument for Separate Analyses of Attitudes Toward Lesbian, Gay, Bisexual Men, Bisexual Women, MtF and FtM Transgender Individuals. Sex Roles. 2013;68(11–12):703–23. 10.1007/s11199-012-0155-1. WOS:000320706500007.

[37] Yost MR, Thomas GD. Gender and binegativity: Men's and women's attitudes toward male and female bisexuals. Archives of Sexual Behavior. 2012;41(3):691–702. 10.1007/s10508-011-9767-8. WOS:000303508500022. 21597943

[38] Herek GM. Heterosexuals' Attitudes toward Bisexual Men and Women in the United States. The Journal of Sex Research. 2002;39:264–74. 10.1080/00224490209552150 12545409

[39] Flores AR, Barclay S. Public support for marriage for same-sex couples by state. The Williams Institute: UCLA, 2013.

[40] Dodge B, Schick V, Friedman MR, Herbenick D, Reece M. Bisexualities: Indiana Attitudes Scale. PsycTESTS: 2014.

[41] Reece M, Herbenick D, Schick V, Sanders SA, Dodge B, Fortenberry JD. Background and considerations on the National Survey of Sexual Health and Behavior (NSSHB) from the investigators. J Sex Med. 2010;7 Suppl 5:243–5. Epub 2010/11/05. 10.1111/j.1743-6109.2010.02038.x . 21029382

[42] Herbenick D, Reece M, Schick V, Sanders SA, Dodge B, Fortenberry JD. Sexual Behavior in the United States: Results from a National Probability Sample of Men and Women Ages 14–94. The Journal of Sexual Medicine. 2010;7:255–65. 10.1111/j.1743-6109.2010.02012.x 21029383

[43] Barker MRC, Jones R, Bowes-Catton H, Plowman T, Yockney J, Morgan M. The bisexuality report: Bisexual inclusion in LGBT equality and diversity. The Open University Centre for Citizenship, Identities and Governance, 2012 Contract No.: 978-1-78007-414-6.

[44] Galupo MP, Davis KS, Grynkiewicz AL, Mitchell RC. Conceptualization of Sexual Orientation Identity Among Sexual Minorities: Patterns Across Sexual and Gender Identity. Journal of Bisexuality. 2014;14(3–4):433–56. 10.1080/15299716.2014.933466

[45] Mitchell RC, Davis KS, Galupo MP. Comparing perceived experiences of prejudice among self-identified plurisexual individuals. Psychology & Sexuality. 2015;6(3):245–57. 10.1080/19419899.2014.940372

[46] Pew Research Center. Support steady for same-sex marriage and acceptance of homosexuality 2016 [cited 2016 June 13]. Available: http://www.pewresearch.org/fact-tank/2016/05/12/support-steady-for-same-sex-marriage-and-acceptance-of-homosexuality/.

[47] Nadler JT, Weston R, Voyles EC. Stuck in the middle: the use and interpretation of mid-points in items on questionnaires. The Journal of general psychology. 2015;142(2):71–89. Epub 2015/04/04. 10.1080/00221309.2014.994590 . 25832738

[48] Garland R. The mid-point on a rating scale: Is it desirable. Marketing Bulletin. 1991;1(1):66–70.

[49] Miller WB, Jones J, Pasta DJ. An implicit ambivalence-indifference dimension of childbearing desires in the National Survey of Family Growth. Demographic Research. 2016;34(7):203–42.

[50] Anselmi P, Vianello M, Voci A, Robusto E. Implicit Sexual Attitude of Heterosexual, Gay and Bisexual Individuals: Disentangling the Contribution of Specific Associations to the Overall Measure. PLoS ONE. 2013;8(11):e78990 10.1371/journal.pone.0078990 24260142 PMC3832517

[51] Parker K. Among LGBT Americans, bisexuals stand out when it comes to identity, acceptance. Pew Research Center, 2015.

[52] Friedman MR, Wood BL, Matthews DD, Stall RD. Who you are, or what you do? Associations between psychosocial health disparities, bisexual behavior, and bisexual identity among Black men in the United States. Annual Meeting of the International Academy of Sex Research; Malmö, Sweden 2016.

[53] Dodge B, Jeffries WLt, Sandfort TG. Beyond the Down Low: sexual risk, protection, and disclosure among at-risk Black men who have sex with both men and women (MSMW). Arch Sex Behav. 2008;37(5):683–96. Epub 2008/05/31. 10.1007/s10508-008-9356-7 ; PubMed Central PMCID: PMCPmc2566750. 18512140 PMC2566750

[54] Jeffries WLt. Beyond the bisexual bridge: sexual health among U.S. men who have sex with men and women. American journal of preventive medicine. 2014;47(3):320–9. Epub 2014/06/28. 10.1016/j.amepre.2014.05.002 . 24970239

[55] Friedman MR, Wei C, Klem ML, Silvestre AJ, Markovic N, Stall R. HIV infection and sexual risk among men who have sex with men and women (MSMW): a systematic review and meta-analysis. PLoS One. 2014;9(1):e87139 10.1371/journal.pone.0087139 24498030 PMC3907399

[56] Friedman MR, Plankey MW, Shoptaw S, Herrick AL, Surkan PJ, Teplin L, et al. Bi Now, Bi Later: Stability of Bisexual Behavior and Extent of Viral Bridging Behavior Among Men Who Have Sex with Men and Women (MSMW. Archives of Sexual Behavior. In press. 10.1007/s10508-016-0863-7 PMC5438760 27873033

[57] Herek GM. Heterosexuals' attitudes toward lesbians and gay men: Correlates and gender differences. Journal of sex research. 1998;25(4):451–77.

[58] Pratto F, Stallworth LM, Sidanius J. The gender gap: Differences in political attitudes and social dominance orientation. British Journal of Social Psychology. 1997;36(1):49–68. 10.1111/j.2044-8309.1997.tb01118.x 9114484

[59] Worthen MGF. An Argument for Separate Analyses of Attitudes Toward Lesbian, Gay, Bisexual Men, Bisexual Women, MtF and FtM Transgender Individuals. Sex Roles. 2013;68(11):703–23. 10.1007/s11199-012-0155-1

[60] Lerum K, Dworkin SL. "Bad girls rule": an interdisciplinary feminist commentary on the report of the APA Task Force on the Sexualization of Girls. Journal of sex research. 2009;46(4):250–63. Epub 2009/08/07. 10.1080/00224490903079542 . 19657944

[61] Baldwin A, Dodge B, Schick V, Hubach RD, Bowling J, Malebranche D, et al Sexual self-identification among behaviorally bisexual men in the midwestern United States. Arch Sex Behav. 2015;44(7):2015–26. Epub 2014/10/26. 10.1007/s10508-014-0376-1 ; PubMed Central PMCID: PMCPmc4409915. 25344028 PMC4409915

[62] Anderson E. 21st century jocks: Sporting men and contemporary heterosexuality. London: Palgrave Macmillan UK; 2014.

[63] McCormack M Anderson E, Adams A. Cohort effect on the coming out experiences of bisexual men. Sociology. 2014;48(6):1207–23.

[64] Savin-Williams RC, Joyner K, Rieger G. Prevalence and stability of self-reported sexual orientation identity during young adulthood. Arch Sex Behav. 2012;41(1):103–10. Epub 2012/02/04. 10.1007/s10508-012-9913-y . 22302504

[65] Malebranche DJ. Bisexually active Black men in the United States and HIV: acknowledging more than the "Down Low". Arch Sex Behav. 2008;37(5):810–6. Epub 2008/05/29. 10.1007/s10508-008-9364-7 . 18506612

